# Rapid detection of *Babesia motasi* responsible for human babesiosis by cross-priming amplification combined with a vertical flow

**DOI:** 10.1186/s13071-020-04246-4

**Published:** 2020-07-29

**Authors:** Jinming Wang, Shandian Gao, Shangdi Zhang, Xin He, Junlong Liu, Aihong Liu, Youquan Li, Guangyuan Liu, Jianxun Luo, Guiquan Guan, Hong Yin

**Affiliations:** 1 State Key Laboratory of Veterinary Etiological Biology, Key Laboratory of Veterinary Parasitology of Gansu Province, Lanzhou Veterinary Research Institute, Chinese Academy of Agricultural Science, Xujiaping 1, Lanzhou, Gansu 730046 People’s Republic of China; 2 Department of Clinical Laboratory, The Second Hospital of Lanzhou University, Lanzhou, Gansu 730000 People’s Republic of China; 3 Jiangsu Co-Innovation Center for the Prevention and Control of Important Animal Infectious Disease and Zoonoses, Yangzhou University, Yangzhou, 225009 People’s Republic of China

**Keywords:** Ovine babesiosis, Human babesiosis, *Babesia motasi*, Cross-priming amplification, Vertical flow visualization strip, Detection, Identification

## Abstract

**Background:**

*Babesia motasi* is known as an etiological agent of human and ovine babesiosis. Diagnosis of babesiosis is traditionally performed by microscopy, examining Giemsa-stained thin peripheral blood smears. Rapid detection and accurate identification of species are desirable for clinical care and epidemiological studies.

**Methods:**

An easy to operate molecular method, which requires less capital equipment and incorporates cross-priming amplification combined with a vertical flow (CPA-VF) visualization strip for rapid detection and identification of *B. motasi*.

**Results:**

The CPA-VF targets the *18S* rRNA gene and has a detection limit of 50 fg per reaction; no cross reaction was observed with other piroplasms infective to sheep or *Babesia* infective to humans. CPA-VF and real-time (RT)-PCR had sensitivities of 95.2% (95% confidence interval, CI 78.1–99.4%) and 90.5% (95% CI 72–97.6%) and specificities of 95.8 (95% CI 80.5–99.5%) and 97.9 (95% CI 83.5–99.9%), respectively, *versus* microscopy and nested (n) PCR combined with gene sequencing. The clinical performance of the CPA-VF assay was evaluated with field blood samples from sheep (*n* = 340) in Jintai county, Gansu Province, and clinical specimens (*n* = 492) obtained from patients bitten by ticks.

**Conclusions:**

Our results indicate that the CPA-VF is a rapid, accurate, nearly instrument-free molecular diagnostic approach for identification of *B. motasi*. Therefore, it could be an alternative technique for epidemiological investigations and diagnoses of ovine and/or human babesiosis caused by *B. motasi*, especially in resource-limited regions. 
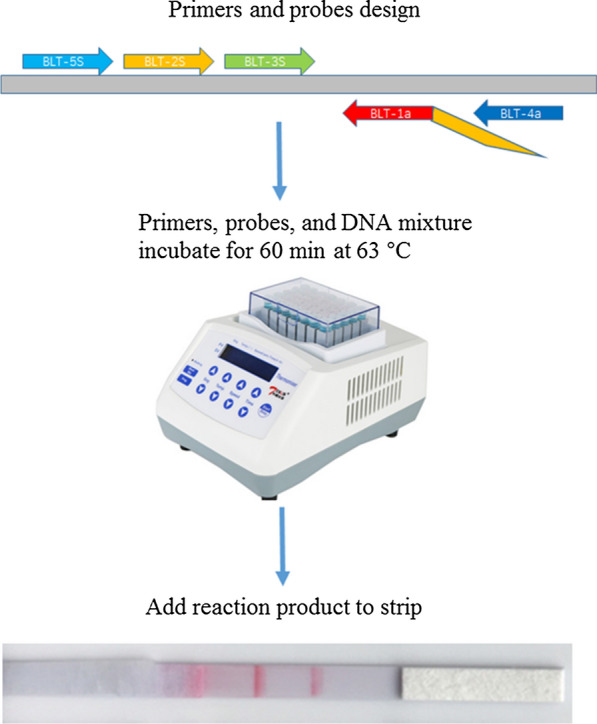

## Background

Babesiosis, caused by protozoan pathogens of the genus *Babesia* infective to humans, domestic and wild animals, is one of the emerging and re-emerging tick-borne disease in the tropical and subtropical regions of the world [1]. It causes a wide spectrum of clinical signs which range from mild fever to serve anemia, haemoglobinuria and even death. Given increasing reports of human babesiosis, great attention has been paid to this emerging human disease [2, 3]. Predominately, three *Babesia* spp., *Babesia microti*, *B. divergens* and *B. duncani*, have been described to be involved in human infections in the USA, Europe and Asia [4, 5]. Recently, two newly emerging *Babesia* species, named as *B. motasi* and *B. crassa*, which were previously reported as causative agents of ovine babesiaosis, have been sporadically reported in cases of human babesiosis in Asia [6–10]. As a causative agent responsible for human babesiosis, the first case caused by *B. motasi*-like was reported in Korea in 2005 [6]. Recently, a 70-year-old man in Korea was diagnosed as infected with *B. motasi* [7].

In China, four strains of *B. motasi* (*B. motasi* Lintan, *B. motasi* Tianzhu, *B. motasi* Ningxian and *B. motasi* Hebei) are responsible for ovine babesiosis, and have been isolated from different endemic areas by the Vector and Vector-Borne Diseases (VVBD) Laboratory, Lanzhou Veterinary Research Institute (LVRI) [11–13]. Epidemiological studies have revealed that *B. motasi* infections have a wide distribution in sheep, goats, and vector ticks across China, according to molecular detection and serological analysis [14–16]. Given that it poses a severe threat to public health, rapid and accurate detection of *B. motasi* infection is important for performing epidemiological studies and providing appropriate clinical management. Several methods, based on molecular techniques that detect the presence of *B. motasi* genomic DNA, have been extensively accepted as the usual strategies for diagnosis of *B. motasi* infection. These methods, including polymerase chain reaction (PCR), RT-PCR, reverse line blot (RLB), and loop-mediated isothermal amplification (LAMP), require costly instruments and skilled personnel to perform the procedures, which has restricted their wide application in clinical care, infection control, and epidemiological studies [15, 17, 18].

Cross-priming amplification (CPA), a novel isothermal amplification technique, was developed as an alternative methodology for disease diagnosis in endemic areas where limited resources were available [19]. This approach has been applied to detection of a number of animal and plant pathogens, such as bacteria, viruses, and herbal products, with high specificity and sensitivity [20–23]. Given that it is an effective detection technique for reliable diagnosis of pathogen infection, in the present study a novel CPA targeting the *18S* rRNA gene was established for on-site detection of *B. motasi* infection. The labeled products from the CPA can be detected using a VF strip to visualize the specific amplicon of *B. motasi*.

## Methods

### Primer design

*Babesia motasi* specific primers for CPA were designed using the sequence alignments of the *18S* rRNA gene of *Babesia* spp. and *Theileria* spp. infective for sheep and humans (Table 1). A region that is conserved intra-*B. motasi* and variable among species was used as the target sequence for primer location. Two sets of primers and probes were designed using Primer Premier 5.0 software (Premier Biosoft International, Palo Alto, CA, USA); each set of primers and probes was composed of two displacement primers (BLT-5 s and BLT-4a), one cross primer (BLT-2 s1a), and two detector primers (BLT-2s and BLT-3 s). The detector primer (BLT-2 s) was labeled with biotin at the 5′-end and the BLT-3 s was labeled with fluorescein isothiocyanate (FITC) at the 5′-end. The cross primer was composed of the BLT-2 s at the 5′-end and 1a at the 3′-end. These primers were synthesized by TsingKe Biotech Co., Ltd (Beijing, China).

**Table 1:** The sequences of *B. motasi* CPA-VF primers and probes

	Primer name	Sequence (5′-3′)
Set one	BLT-5 s	GCTAATTGTAGGGCTAATACAAG
	BLT-2 s	FITC-CGATGCCTTTTGGCGGCG
	BLT-3 s	Biotin-GCTTTTAAACCAATTGTTGG
	BLT-2s1a	CGATGCCTTTTGGCGGCGCGATTCGCAAGTTTATTATG
	BLT-4a	CTTGAATGGAACATCGCTAA
Set two	BLT-5 s	GGADWWDGTCCGKTTTTG
	BLT-2 s	FITC-CTTAGAGGGACTCCTGC
	BLT-3 s	Biotin-GCTTGAAGCGTGGGGT
	BLT-2s1a	CTTAGAGGGACTCCTGCCAGACCTGTTATTGCCTT
	BLT-4a	CGCCTGCCGTTCGACGATT

### Blood samples

Standard positive samples were obtained from sheep experimentally infected with *B. motasi*. Briefly, 16 6-month-old sheep that were divided into 4 groups (groups 1–4) containing the same number of experimental animals were purchased from Jingtai county, Gansu Province, China, and confirmed to be free of piroplasm infection by microscopy, RT-PCR, nPCR and ELISA assay [14, 18, 24, 25]. Groups 1–4 were inoculated intravenously 10 ml of cryopreserved blood infected with *B. motasi* Lintan, *B. motasi* Tianzhu, *B. motasi* Ningxian and *B. motasi* Hebei, respectively. When parasitemia reached 8–10%, blood samples were collected into EDTA-coated tubes. Three intact sheep were inoculated with 50 ml blood infected with either *B. motasi* Lintan/*B. motasi* Tianzhu/*B. motasi* Ningxian/*B. motasi* Hebei via the jugular vein. Blood from the jugular vein was collected every 2 days after *Babesia* inoculation. Negative blood samples were collected into EDTA coated tubes from randomly selected sheep in Jintai county, Gansu province, where *B. motasi* is not endemic. All blood samples were transported to the VVBD laboratory, LVRI in iceboxes and stored at −20 °C before DNA extraction.

Genomic DNA was extracted from 200 μl of the above-mentioned blood samples using a commercial DNA extractions kit according to the manufacturer’s instruction (QIAamp DNA Blood Mini Kit; Qiagen, Hilden, Germany).

### Optimization of the CPA-VF assay for *B. motasi* detection

Initially, we designed two sets of primers to develop a highly sensitive and specific method. The CPA amplification was performed in a final volume of 20 μl. Following optimization of the reaction, the final composition was as follows: 1.25 μM each of displacement primer (BLT-5 s and BLT-4a); 7.5 μM each of detector primer (BLT-2 s and BLT-3 s); 12.5 μM of cross primer (BLT-2s1a); 6 mM MgSO_4_, 20 mM Tris–HCl (pH 8.8); 10 mM KCl; 1 M betaine; 8U *Bst* DNA polymerase (New England BioLabs, Ipswich, UK); 8 mM deoxynucleotides triphosphates (dNTPs); 0.1% Triton X-100; and 2 μl genomic DNA. The CPA reaction tubes were incubated at 63 °C for 60 min, followed by 80 °C for 2 min to terminate the reaction. Finally, VF strips, purchased from Hangzhou Ustar Company (Hangzhou, China), were used to detect CPA products: 5 μl of CPA products and 90 μl of PBS were added to the sample pad. A reaction was identified as positive when both the test line and the control line were developed, whereas it was considered as negative when only the control line was developed.

Furthermore, CPA reactions were performed at different temperatures, ranging from 55 °C to 65 °C, and various time settings, ranging from 40 to 100 min. Subsequently, the amplified products were detected using VF strips.

### Specificity and sensitivity of the CPA assays

Genomic DNAs of *Theileria luwenshuni*, *T. uilenbergi*, *T. ovis*, *Babesia* sp. Xinjiang and *Babesia* sp. Dunhuang were provided by VVBD. The specificity of the assay was evaluated using genomic DNA from *B. motasi* Lintan, *B. motasi* Tianzhu, *B. motasi* Ningxian, *B. motasi* Hebei, *Babesia* sp. Xinjiang, *Babesia* sp. Dunhuang, *T. luwenshuni*, *T. uilenbergi*, *T. ovis*, *B. divergens*, *B. duncani*, and plasmid DNAs bearing the *18S* rRNA gene of *B. microti* (GenBank: KF410825) and *B. crassa* (GenBank: AY260176). To evaluate the assay’s sensitivity, serial dilutions of genomic DNA from purified *B. motasi* Lintan merozoites were used as the template for CPA amplification using the following concentrations: 4 ng/μl; 800 pg/μl; 160 pg/μl; 32 pg/μl; 6.4 pg/μl; 1.28 pg/μl; 0.256 pg/μl; 50 fg/μl; and 10 fg/μl. Each concentration of genomic DNA was tested in three independent experiments to ensure reproducibility of the CPA assay.

To evaluate the performance of the CPA-VF assay, its sensitivity and specificity were determined using standard positive samples (experimentally infected animals) and field collected negative samples, *versus* microscopy, RT-PCR, and nPCR targeting the *18S* rRNA combined with gene sequencing [18, 24, 26].

### Clinical performance of the CPA-VF assay for clinical specimens

Field blood samples were randomly collected from 340 sheep in Gansu Province, transported to VVBD, LVRI in iceboxes and stored at −20 °C before DNA extraction. A total of 492 patients who lived in the Gannan Tibetan Autonomous Prefecture (Gansu Province) who had visited the Second Hospital of Lanzhou University for a tick bite in the past few months, between May 2017 and July 2019, were recruited. Blood samples collected from patients were tested using the CPA-VF assay to determine the infection status of *B. motasi.*

The clinical performance of the CPA-VF approach was evaluated with field blood samples collected from sheep and clinical specimens from patients with a history of tick bite.

## Results

### Optimization of the CPA primers, reaction temperature and time

**Fig. 1:**
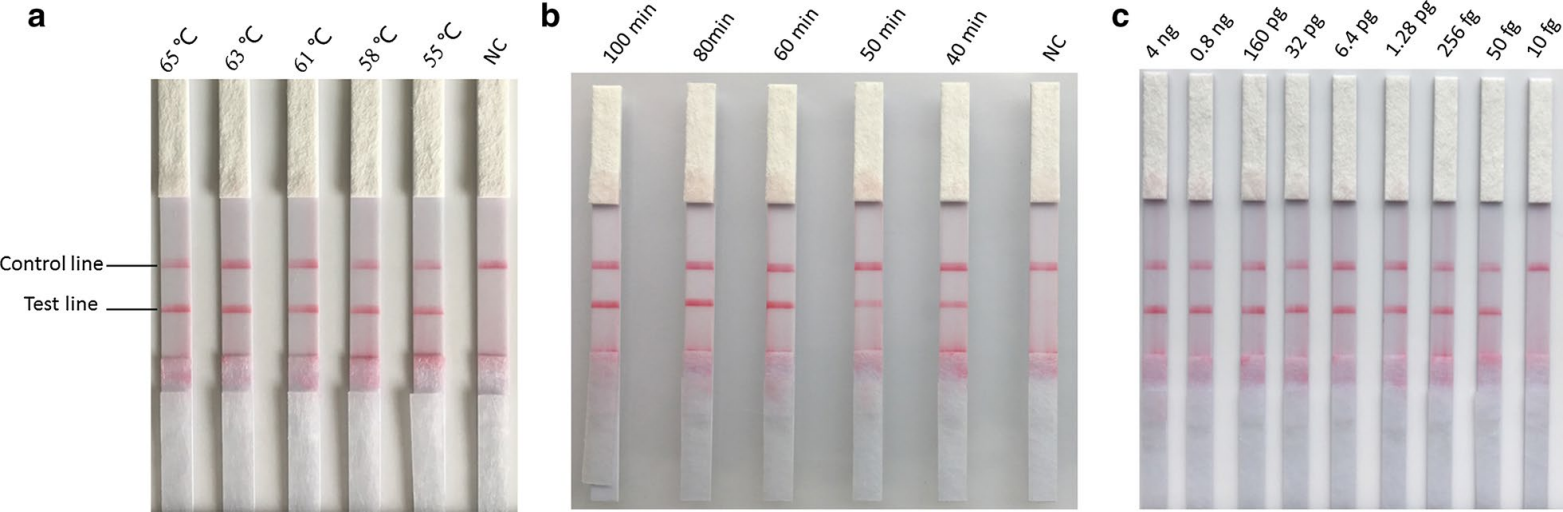
Optimization of CPA reaction temperature and time and limit of detection evaluation of the CPA-VF assay with serial dilution of *B. motasi* DNA. **a** Reaction temperature of CPA-VF assay. **b** Reaction time of CPA-VF assay. **c** Detection limit of the CPA-VF

Sequence alignment of the *18S* rRNA genes of piroplasms infective to sheep and goats available in NCBI showed that two regions are conserved intra-species and variable among species. The sequences and locations of the primers are presented in Table 1. The primers and probes of set one showed high specificity. Therefore, set one was used for subsequent experiments. To determine a suitable amplification temperature, the CPA reactions were incubated at 55–65 °C for 60 min. The results showed that the assay could be performed at a wide range of temperatures, from 58 to 63 °C. Changes in amplification temperature had a slight impact on the brightness of the bands, indicating that incubation temperature is significant for the CPA reaction. The optimal brightness of a red–purple band was observed in the VF strip at a temperature 61 °C (Fig. 1a).

The CPA amplification was conducted at 61 °C for 40–100 min. The results revealed that positive signs could be developed as early as 40 min after amplification; however, the brightness of the positive band was not strong as after 60 min and 80 min. To provide a high sensitivity and time efficiency of the CPA assay, an amplification time of 60 min was used in *B. motasi* detection (Fig. 1b).

### Cross reaction of the developed CPA approach

**Fig. 2:**
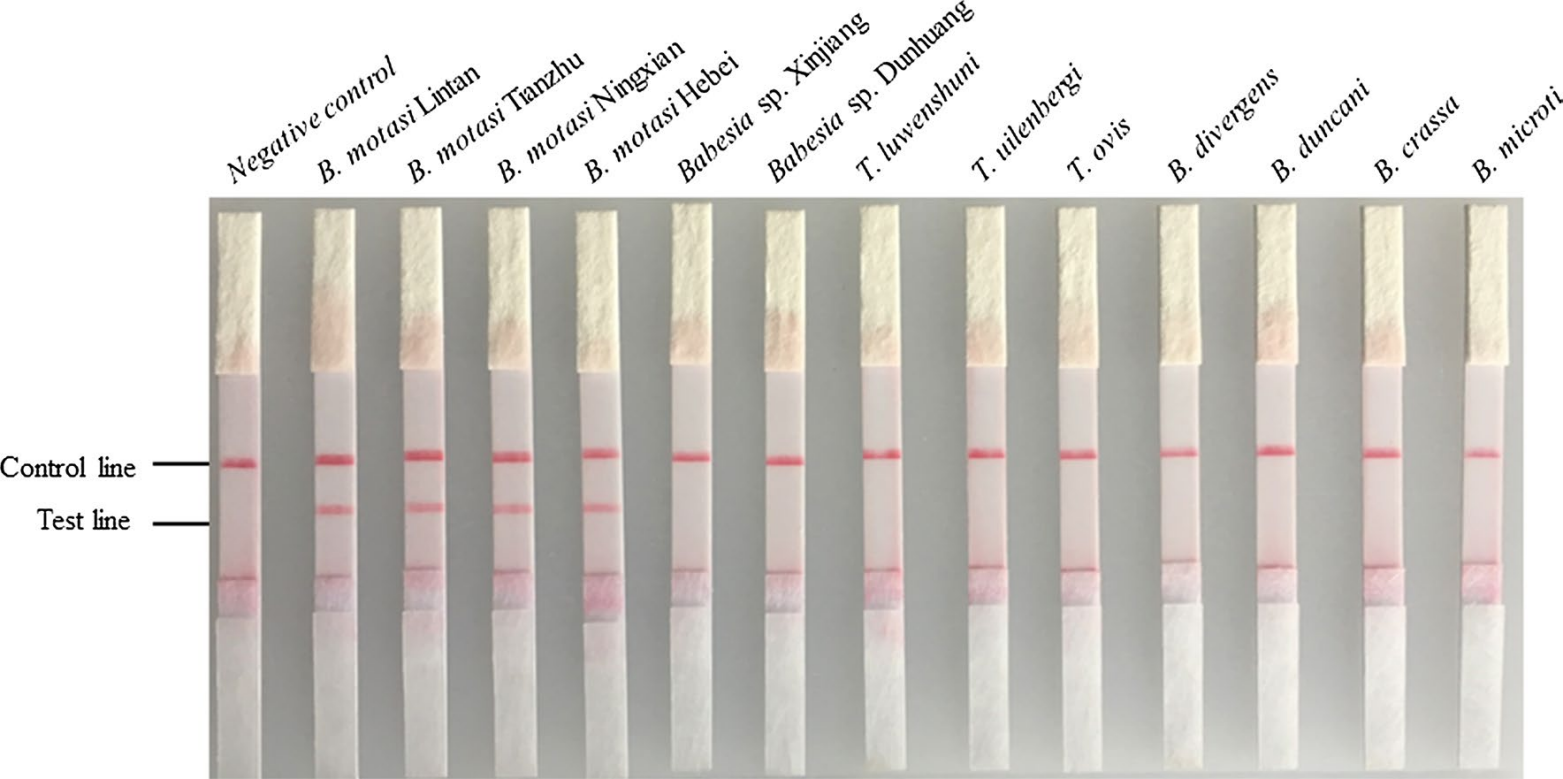
Evaluation of specificity of the CPA-VF with genomic DNA (*B. motasi* Lintan, *B. motasi* Tianzhu, *B. motasi* Hebei, *B. motasi* Ningxian, *Babesia* sp. Xinjiang, *Babesia* sp. Dunhuang, *T. uilenbergi*, *T. luwenshuni*, *T. ovis*, *A. ovis*, *B. duncani* and *B. divergens*) and plasmids (*B. microti* and *B. crassa*)

The CPA technique was evaluated by testing piroplasms infective for sheep, goats and humans. As shown in Fig. 2, no cross reaction was observed with other *Babesia* spp. and *Theileria* spp. These results demonstrated that the CPA assay is specific for identification of *B. motasi* (Fig. 2).

### Limit of detection of the CPA-VF assay

The limit of detection of the CPA-VF assay was evaluated using fivefold serially diluted DNA from purified merozoites of *B. motasi* in three independent reactions from 4 ng/μl to 10 fg/μl. The assay could detect as few as 50 fg/μl DNA of *B. motasi* (Fig. 1c). As shown, two bands on the VF strips were developed using 4 ng to 50 fg of genomic DNA, while only one band was observed with 10 fg and negative control reactions.

### Sensitivity and specificity of CPA-VF

A total of 42 samples of standard positive genomic DNA from experimentally infected sheep and 48 negative field samples were studied using both CPA-VF and RT-PCR (Table 2). The performance of CPA-VF and RT-PCR is presented in Table 3. The CPA-VF and RT-PCR had sensitivities of 95.2% (95% confidence interval, CI 78.1–99.4%) and 90.5% (95% CI 72–97.6%) and specificities of 95.8% (95% CI 80.5–99.5%) and 97.9% (95% CI 83.5–99.9%) (Table 3). There was no significance difference between the performance of the CPA-VF and RT-PCR methods.

**Table 2:** Standard positive and negative samples, confirmed by thin blood smear microscopy and nested PCR combined with gene sequencing

Result	*B. motasi* Lintan (*n*)	*B. motasi* Tianzhu (*n*)	*B. motasi* Hebei (*n*)	*B. motasi* Ningxian (*n*)	Negative (*n*)	Total (*n*)
Positive	10	10	12	10		42
Negative					48	48
Total	10	10	12	10	48	90

*n* number of samples

**Table 3:** The performance of the CPA-VF assay compared with that of RT-PCR

Result	Detection method	
	RT-PCR	CPA-VF
True positive	38	40
False positive	1	2
True negative	47	46
False negative	4	2
Sensitivity (%)	90.5 (72–97.6)	95.2 (78.1–99.4)
Specificity (%)	97.9 (83.5–99.9)	95.8 (80.5–99.5)

### Evaluation of the CPA using samples from the field and clinical samples

To evaluate the feasibility of using CPA-VF as an alternative approach for *B. motasi* detection, 340 whole blood samples from sheep and patients were subjected to CPA-VF. The results of the CPA-VF assay showed that 3.8% (13/340) of the samples collected from sheep in Gansu province were positive and the remaining samples were negative for *B. motasi* infection.

A total of 492 blood samples collected from patients bitten by ticks, who visited the hospital, were investigated for the presence of *B. motasi*. From the results of CPA-VF, three samples were positive for *B. motasi* infection. Furthermore, to validate the presence of *B. motasi* in these samples, RT-PCR and nPCR were also employed and the results showed that all samples were negative for *B. motasi* infection.

## Discussion

To provide an effective diagnostic tool, a CPA method targeting the *18S* rRNA sequences of *B. motasi* was successfully developed for rapidly detecting and discriminating *B. motasi* infection. The CPA assay could detect four strains of *B. motasi*: *B. motasi* Lintan, *B. motasi* Tianzhu, *B. motasi* Hebei, and *B. motasi* Ningxian. In addition, no cross-reaction was observed with piroplasms infective for sheep (*Babesia* sp. Xinjiang, *T. uilenbergi*, *T. luwenshuni*, *T. ovis* and *A. ovis*) and humans (*B. duncani*, *B. divergens*, *B. microti* and *B. crassa*). Further studies should be performed to investigate any potential cross-reactivity with other pathogens infective for humans using the CPA-VF approach developed herein.

In our present study, CPA-VF could detect as few as 50 fg of genomic DNA from *B. motasi* per reaction, which was equal to approximately 50 μl of 0.000,005% parasitized erythrocytes. The CPA reaction does not require expensive equipment and can be performed in a constant temperature block to maintain a reaction temperature of 61 °C for 60 min. Furthermore, products generated by the CPA amplification can be detected using a VF strip, which only takes 2–5 min and is visible to the naked eye. Thus, the CPA assay is suitable for rapid, simple, and sensitive detection of *B. motasi* infection in limited-resource settings in endemic regions.

To assess its suitability for clinical use, we conducted the first diagnostic study in clinical specimens and host animals using CPA-VF, comparing it with microscopy, RT-PCR, and nPCR combined with PCR product sequencing. The results from studies of a positive and negative panel revealed that CPA-VF has better sensitivity than that of RT-PCR. Because of its sensitivity, CPA-VF could be useful for the preliminary screening of low-level parasitemia. Our results demonstrate that excellent sensitivity was observed for the CPA-VF approach in comparison with that of RT-PCR. However, false positives that needed to be confirmed by microscopy should be noted with the CPA-VF assay. Three samples determined to be negative for piroplasm infection by nPCR were shown to present *B. motasi* infections by CPA-VF analysis. According to the clinical records, these three people presented virus-like or flu-like symptoms, clinical laboratory data showed they might be infected with bacteria (Additional file 1: Table S1). They were treated with azithromycin and cephalosporin and did not come back to hospital again. As these cases were outpatients we were not able to obtain enough information about the outcome of these patients. One limitation of this study was the small number of positive specimens, which were used to evaluate clinical performance. Further studies are needed regarding the implementation of this approach into clinical practice.

## Conclusions

We successfully developed CPA-VF analysis for rapid and specific detection of *B. motasi*, with high sensitivity (95.2%) and specificity (95.8%). The developed CPA-VF assay does not require sophisticated equipment and has an easy nucleic acid detection system. The study provided a practical, easy-to-operate and alternative method for performing epidemiological and point-of-care diagnosis for *B. motasi* infection, although there should be some caution regarding false positives when CPA is used for clinical screening.

## Supplementary information

**Additional file 1: Table S1.** Clinical information for three patients.

## Data Availability

Not applicable.

## Abbreviations

CPA-VF: Cross-priming amplification combined with a vertical flow
LAMP: Loop-mediated isothermal amplification
RT-PCR: Real-time PCR
VVBD: Vectors and vector-borne diseases laboratory
LVRI: Lanzhou Veterinary Research Institute
nPCR: Nested PCR
RLB: Reverse line blot
