# Supplementary material for: Rapid detection of Babesia motasi responsible for human babesiosis by cross-priming amplification combined with a vertical flow
Source: Parasit Vectors. 2020 Jul 29;13:377. doi: 10.1186/s13071-020-04246-4 (PMC7391542; doi:10.1186/s13071-020-04246-4)
Supplement: Supplementary file 1 — Additional file 1: Table S1. Clinical information for three patients. [file 13071_2020_4246_MOESM1_ESM.docx]

**Additional file 1: Table S1.** Clinical information for three patients.

| Characteristic | Nos of patients | | |
| --- | --- | --- | --- |
|  | 1 | 2 | 3 |
| Sex | M | F | M |
| Age(year) | 49 | 35 | 55 |
| Clinical manifestation | fever | headache | fever anorexia |
| Leukocyte (10^9^/L) | 5.9 | 5.7 | 7.9 |
| Red blood cells (10^12^/L) | 7.2 | 5.9 | 5.3 |
| Hemoglobin concentration (g/L) | 147 | 172 | 165 |
| Platelet (10^9^/L) | 127 | 193 | 187 |
| Lymphocyte (10^9^/L) | 1.9 | 2.3 | 1.7 |
| Neutrophil (10^9^/L) | 3.6 | 2.7 | 3.8 |
| Hospital stay | NO | NO | NO |

Reference ranges: Leukocyte count, 3.97-9.15×10^9^/L; Red blood cells count, 4.09-5.74×10^12^/L; Hemoglobin concentration, 131-172 g/L; Platelet count, 85-303×109/L; Lymphocytes count, 0.8-4×10^9^/L Neutrophils count, 1.8-6.3×10^9^/L.
